# A smoking quitline integrated with clinician counselling at outpatient health facilities in Vietnam: a single-arm prospective cohort study

**DOI:** 10.1186/s12889-022-13203-y

**Published:** 2022-04-13

**Authors:** Wan-Chun Huang, Guy B. Marks, Ngoc Yen Pham, Thu Anh Nguyen, Thuy Anh Nguyen, Van Giap Vu, Viet Nhung Nguyen, Stephen Jan, Joel Negin, Quy Chau Ngo, Greg J. Fox

**Affiliations:** 1 Woolcock Institute of Medical Research, Hanoi, Vietnam; 2 South Western Sydney Clinical School, The University of New South Wales, Sydney, Australia; 3 Respiratory Center, Bach Mai Hospital, Hanoi, Vietnam; 4 National Tuberculosis Control Program of Vietnam, Hanoi, Vietnam; 5 Health Economics and Process Evaluation Program, The George Institute for Global Health, Sydney, Australia; 6 School of Public Health, The University of Sydney, Sydney, Australia; 7 Faculty of Medicine and Health, The University of Sydney, Sydney, Australia

**Keywords:** Smoking cessation, Quitline, Text messages, Brief advice

## Abstract

**Background:**

Limited evidence is available about the combination of multiple smoking cessation modalities in low- and middle-income countries. The study aimed to assess the feasibility of a smoking cessation intervention that integrates follow-up counselling phone calls and scheduled text messages with brief advice from physicians in Vietnam.

**Methods:**

This was a single-arm intervention study. Smokers were referred to the study Quitline after brief advice by physicians at three rural district hospitals in Hanoi, Vietnam. Following referral, participants received nine counselling phone calls in 12 months and a scheduled text message service that lasted for three months. Participants who reported smoking cessation for at least 30 days at the 12-month follow-up were invited for a urinary cotinine test to confirm cessation.

**Results:**

The Quitline centre had 431 referrals from participating hospitals. Among them, 221 (51.3%) were enrolled. After the baseline phone call, 141 (63.8%) participated in all 4 follow-up calls within the first month and 117 (52.9%) participated in all phone calls in 12 months. The median number of successful phone calls was 8 (interquartile range: 6 – 8). At the end of the study, 90 (40.7%) self-reported abstinence from smoking over the previous 30 days. Among them, 22 (24.4%) submitted a sample for cotinine test, of which 13 (59.1% of those tested) returned a negative result. The proportion of biochemically-verified quitters was 5.9%.

**Conclusions:**

The integration of brief advice and referral from healthcare facilities, Quitline counselling phone calls, and scheduled text messaging was feasible in rural health facilities in northern Vietnam.

**Trial registration:**

ACTRN12619000554167.

**Supplementary Information:**

The online version contains supplementary material available at 10.1186/s12889-022-13203-y.

## Background

Tobacco smoking remains a leading cause of premature death and chronic diseases worldwide. Despite the abundant evidence to assist people quit smoking, poor reach and utilisation of smoking cessation interventions has been observed in many low and middle-income countries (LMICs) [1, 2].

Numerous barriers prevent the scale-up of smoking cessation interventions in resource-limited settings [3, 4]. These include competing time pressures and inadequate counselling skills of healthcare workers, personal tobacco use by doctors and a lack of support from senior clinical leaders [5]. Just one third of middle-income countries, and almost no low-income countries, have established telephone Quitlines [6]. Furthermore, nicotine replacement and other smoking cessation therapies are often unaffordable or unavailable [7].

In Vietnam, tobacco control within health sector remains a challenge, despite high-level government commitment. Directive 05/CT-BYT was issued in 2013 to strengthen the implementation of tobacco control activities within the public health sector [8]. The directive requires all public hospitals to implement a smoke-free program within their facilities. This includes prohibition of tobacco selling and a complete ban of smoking within healthcare facilities, including indoor and outdoor spaces. The directive also directs healthcare workers to provide counselling to patients and their family members about tobacco harm and methods of smoking cessation. However, limited data are available regarding the enforcement of smoke-free environment and smoking cessation intervention in Vietnamese healthcare facilities. Evidence regarding the feasibility of smoking cessation advice by healthcare workers is also limited.

Despite these challenges, recent evidence demonstrates the feasibility and effectiveness of low-cost smoking cessation interventions in LMICs. Such interventions include cytisine [9, 10], integrating brief advice into other existing healthcare services [3, 11], and text messaging for smokers [12, 13]. However, limited evidence is available about implementing these interventions in LMICs, including the combination of multiple cessation modalities.

The primary objective of the study was to assess the feasibility of a smoking cessation intervention that integrates brief advice from physician, follow-up counselling phone calls, and scheduled text messages within the Vietnamese health system. The secondary objective was to determine biochemically-verified quit rate among participants 12 months after enrolment.

## Methods

### Study design and setting

This single-arm intervention study was conducted in three rural district hospitals in Hanoi, Vietnam.

Healthcare in Vietnam is delivered through four levels of health facility: central (national) hospitals, provincial hospitals, district hospitals, and commune health centres. District hospitals deliver healthcare to their local communities, typically providing both inpatient and outpatient care [14]. Outpatient clinics at district hospitals provide general consultations with basic blood tests and X-rays available on site.

Currently, there are two official toll-free Quitlines supported by Vietnam Tobacco Control Fund. One in northern Vietnam was established in 2015 and run by Bach Mai Hospital, a leading general hospital in Hanoi. The Quitline program is delivered by 10 certified counsellors [15]. The other one in Southern Vietnam was run by Gia Dinh People’s Hospital since 2017.

### Study population and selection criteria

We enrolled patients aged ≥ 12 years presenting to selected district facilities, as well as healthcare workers employed by these facilities. Participants meeting the following criteria were eligible for inclusion: (a) Had smoked at least 100 cigarettes in his/her lifetime, (b) Smoked cigarettes (defined as smoking at least one cigarette in the previous month), (c) Agrees to participate in the smoking cessation programme, (d) Able to communicate effectively, (e) Intends to be resident in the province for the next 12 months.

### Intervention and follow-up

This study evaluated the implementation of a complex intervention. Before enrolment commenced, we engaged with hospital leaders to implement a smoke-free hospital policy, in accordance with national policy and guidelines [16, 17].

Training was provided to healthcare workers about the goals of smoke-free hospitals, and how to deliver brief advice using the ‘5As approach’. Written materials were developed to assist with smoking cessation, based upon health promotion materials from the Ministry of Health in New South Wales, Australia [18, 19]. A Quitline was established at the Hanoi office of Woolcock Institute of Medical Research using the system of a telecommunication company. Quitline counsellors undertook a 3-day training programme by an external expert and on-site training at the Quitline office run by Bach Mai Hospital [15]. Posters with Quitline information were placed in the consultation rooms and public places of the hospitals.

Healthcare workers could refer patients to the Quitline after obtaining informed consent from patients or their legal guardians. People could also refer themselves by calling the toll-free Quitline. Healthcare workers who were current smokers were also invited to join the smoking cessation programme during the training.

The Quitline program included a scheduled one-way text message service that lasted for three months and nine counselling phone calls in 12 months. After each smoker was referred to the study Quitline (i.e. the doctor passed the smoker’s contact info to the study Quitline), the Quitline counsellor called the smoker within 24 h, excluding weekends and public holidays. During this baseline phone call, the Quitline counsellor assessed participants’ eligibility, and enrolled them into the smoking cessation programme. The Quitline counsellor then collected information about study participants, provided counselling and encouraged each smoker to set a planned quit date, preferably within 14 days [20].

After the baseline phone call, the scheduled one-way text messages service started. The Quitline counsellor sent 64 text messages to a participant over a 3-month period. These messages included strategies to avoid smoking cues, deal with cravings, and encouragement (Supplementary Table S1 and Table S2 in Additional file 1 show the schedule and content of the text messages). Quitline counsellor called participants 1 week, 2 weeks, 3 weeks, 4 weeks, 3 months, 6 months, 9 months, and 12 months after baseline to provide cessation counselling. The Quitline operated during working hours (AM 8:30 to PM 5:30). During the calls, the Quitline counsellor asked the participants about the preferred timing for the next call. The text messages were sent out at AM 8:00 on the scheduled dates. The scheduled text messaging service and the Quitline were both free of charge.

### Study outcomes

The primary outcome measure was the proportion of enrolled smokers with biochemically-verified abstinence after 12 months. Individuals who stated they had not smoked in the previous 30 days were asked to submit urine for cotinine testing to verify abstinence. Verification was based upon a test strip that detected the presence of cotinine in urine at a cut-off concentration of 200 ng/mL (Confirm BioSciences, CA. USA). The secondary outcome measures included (a) the proportion of individuals who self-reported not having smoked within the previous 30 days, at the time of the 12-month follow-up, and (b) the proportion of patients reporting at least one quit attempt lasting 30 days during the follow-up period.

Participation in the intervention was evaluated using a “cascade of care” approach. Pre-defined steps in the cascade for those attending health facilities were: (a) the number of smokers attending outpatient health services; (b) the proportion of smokers in the previous step who enrolled in the smoking cessation programme; (c) the proportion of those in the previous step who completed initial outpatient counselling and received smoking cessation material; (d) the proportion of enrolled smokers who reported making at least one quit attempt, lasting at least 30 days, during the 12-month follow-up period; (e) the proportion of enrolled smokers who reported being abstinent from smoking for at least 30 days at 3, 6, 9, and 12 months after enrolment.

Research staff stayed at the registration counter and asked about current smoking status of consecutive patients visiting the health facility during a one-week run-in period. The average number of smokers presenting to the facility per day during this period was then used to estimate the number of smokers at the first step of the cascade.

### Sample size

The sample size was calculated to estimate the proportion of people quitting smoking over a 12-month period. We allowed for 10% loss to follow-up. We had expected to recruit a total of 480 participants, allowing for a 10% incidence of biochemically-verified smoking cessation. This would allow us to estimate the true incidence of smoking cessation within ± 2.8%, given an alpha of 0.05. Owing to difficulties in recruitment, the actual sample size was 221 people. Given an expected incidence of 10% smoking cessation, this sample size allowed us to estimate the quit rate within ± 4.0% of the true value [21].

### Statistical methods

The characteristics of participants were analysed using descriptive statistics. Comparisons of selected baseline demographic characteristics among participants grouped by smoking status at 12 months were performed using Chi-square test and Kruskal–Wallis test to assess a difference in the values of categorical and continuous variables, respectively. An exploratory analysis using multivariable logistic regression was done to identify factors associated with abstinence at 12 months. In the regression, Fagerström Test for Cigarette Dependence was grouped into high (7 – 10), moderate (4 – 6); and low (< 4). The number of successful counselling phone calls was highly negatively skewed, and normalisation was not achieved. We grouped the number of phone calls made per participant as 8 or above, 6 to 7, and less than 6, according to quartiles. A p-value less than 0.05 was considered statistically significant. Analyses were conducted using SAS® (v9.4, SAS Institute, Cary Corp. NC. USA).

### Ethical issues

Healthcare workers obtained informed consent from patients to receive brief counselling and to authorise their phone numbers to be sent to Quitline staff. Additional informed consent by participants or their legal guardians was provided during the initial phone call, to enable data collection, participation in counselling and follow-up. All methods were carried out in accordance with relevant guidelines and regulations. Ethical approval was provided by the Human Research Ethics Committee at the University of Sydney (Protocol 2018/769), and the Institutional Review Board of the Bach Mai Hospital, Hanoi, Vietnam (Approval 3497/QD-BM). The trial was registered with the Australian New Zealand Clinical Trial Registry (ACTRN12619000554167) on 09/04/2019.

## Results

**Fig. 1:**
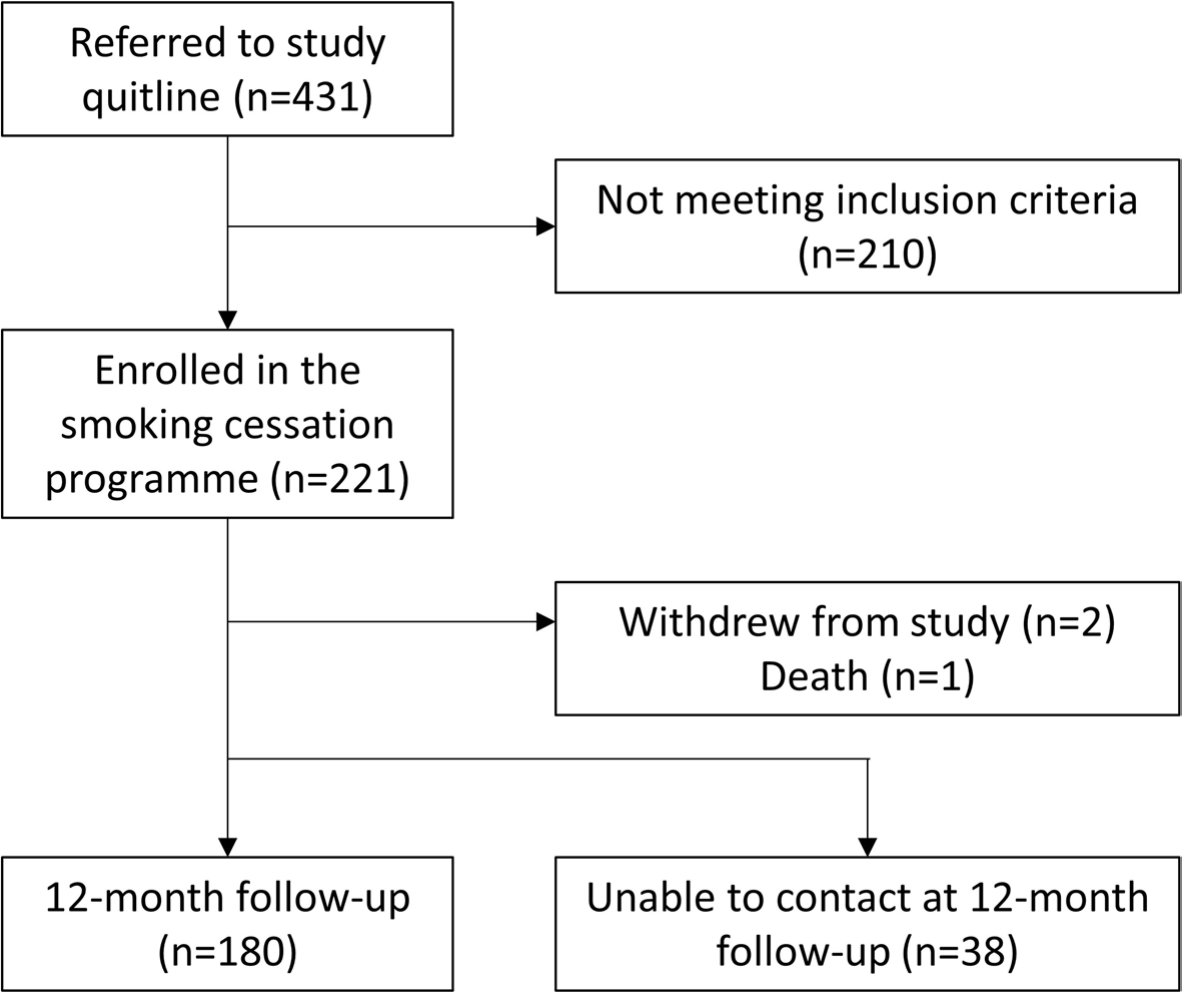
CONSORT diagram of the study

**Fig. 2:**
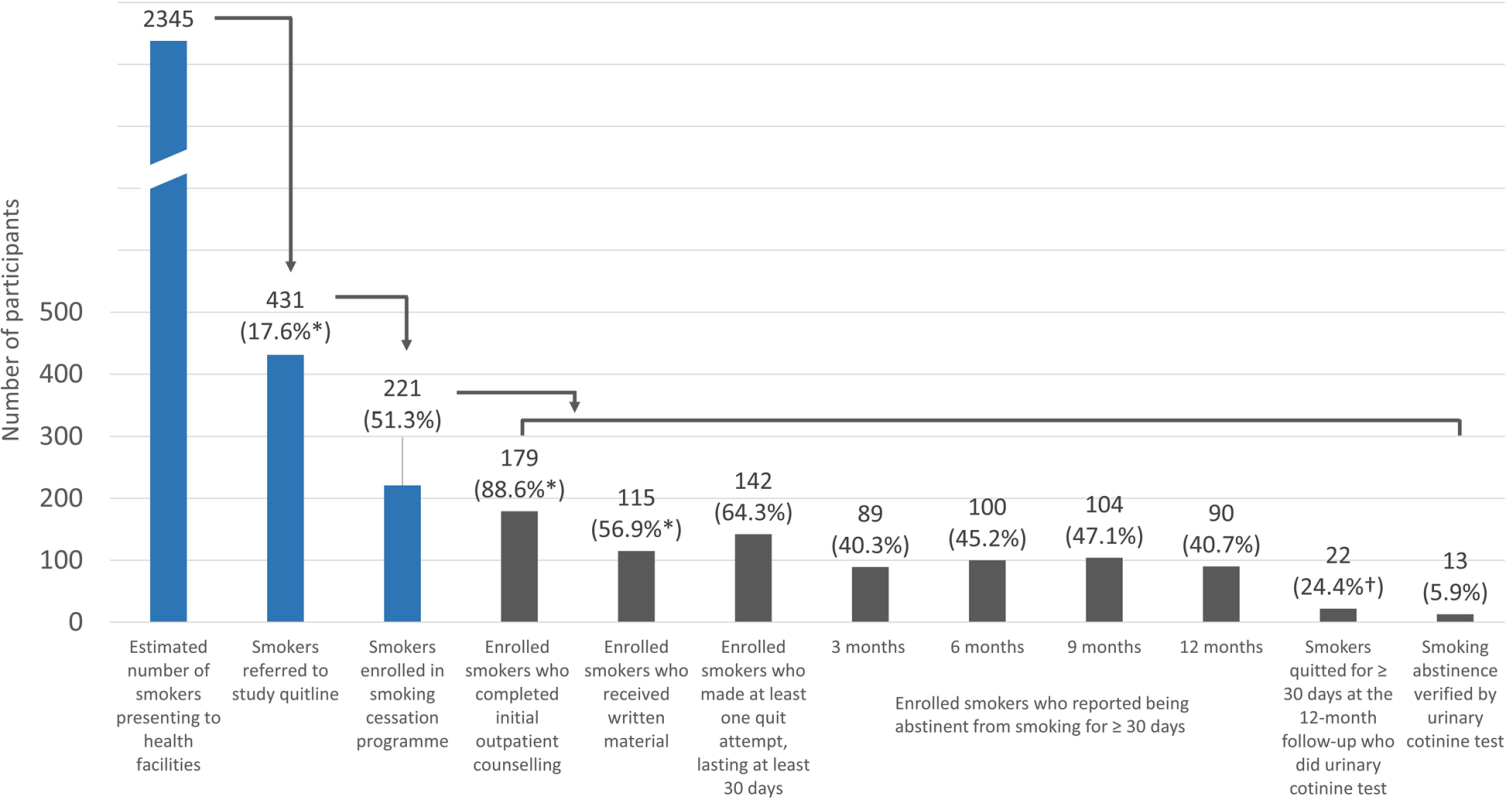
Proportion of smokers presenting to health facilities completing each step of the cascade. *Only patients referred by doctors included in the proportion calculation. †Denominator is the 90 subjects with self-reported smoking abstinence at 12 months

Figure 1 shows the CONSORT diagram for recruitment to the study. Between May 2019 and January 2020, 431 individuals were referred to the Quitline, including 14 patients referred themselves by calling the Quitline, and five healthcare workers (Fig. 2). Based upon estimates obtained from enumeration during the run-in period, about 17.6% of smokers visiting healthcare facilities on the dates of referral were referred to the study Quitline by healthcare workers. Among the 431 referred to the study Quitline, 221 (51.3%) meeting the eligibility criteria agreed to participate in the smoking cessation programme.

Among patients attending the Quitline, 179 (88.6%) reported that they received brief counselling by their doctor, and 115 (56.9%) received written smoking cessation material from the doctor (Fig. 2). During the 12-month follow-up, 142 out of 221 enrolled subjects (64.3%) reported making at least one quit attempt that lasted at least 30 days. At the end of the study, 90 (40.7%) participants self-reported having abstained from smoking for at least the previous 30 days. Among these, 22 (24.4%) agreed to take a urinary cotinine test, of which 13 (59% of those tested) returned a negative result. Overall, the proportion of verified quitters was 5.9% of those enrolled in the smoking cessation programme at 12 months.

Of the five healthcare workers who participated, only one achieved verified smoking cessation. The rest four remained smoking at the end of the study and none of them had a quit attempt that lasted for more than 30 days.

Among the 221 enrolled smokers, 141 (63.8%) answered all 4 counselling phone calls within the first month and 117 (52.9%) answered all the 8 phone calls. The median number of successful phone calls made was 8 (interquartile range: 6 – 8).

Table 1 shows the baseline characteristics of the 221 enrolled smokers. All smokers were men, with a median age of 51 years (interquartile range: 38 – 61 years). The median score on the Fagerström Test for Cigarette Dependence was 6 (interquartile range: 4 – 8). The majority (73.8%) of smokers had attempted to quit at least once previously. Personal health was the main reason they expressed interest in quitting at the present encounter (95.5%). Only 4.1% referred to the expense of smoking as their reason to quit.

**Table 1:** Baseline characteristics of smokers referred to the smoking Quitline

Characteristic	Total (*n* = 221)
**Demographic factors**	
Age, median years (IQR)	51 (38 – 61)
Male sex, n (%)	221 (100)
Highest level of education attained, *n* (%)	
Less than primary	3 (1.4)
Primary	5 (2.3)
Lower secondary	92 (41.6)
Upper secondary	78 (35.3)
University degree, or equivalent, or higher	43 (19.5)
**Smoking-related factors**	
Median average number of cigarettes/day (IQR)^*^	20 (10 – 30)
Score on the Fagerström Test for Cigarette Dependence at baseline (IQR)^*^	6 (4 – 8)
Median years smoking (IQR)	25 (15 – 40)
At least one prior quit attempt, *n* (%)	163 (73.8)
Drink alcohol every day, *n* (%)	83 (37.6)
Drink caffeinated drinks every day, *n* (%)	157 (71.0)
Living with at least one other smoker, *n* (%)	53 (24.0)
Reasons given to quit, *n* (%)	
Personal health condition	211 (95.5)
Family’s health	28 (12.7)
Expense	9 (4.1)

^*^63 missing values

IQR, interquartile range

Table 2 compares the characteristics of participants by their final smoking status. Among all participant characteristics, the initial decision to specify a planned personal quit date was associated with an increase in successful quitting. Participants who set a personal quit date within 14 days from the baseline had a higher chance of quitting (still smoking vs. self-reported cessation, *p* = 0.034; still smoking vs. biochemically-verified cessation, *p* = 0.031).

**Table 2:** Comparison of characteristics by participants’ smoking status at the end of study

Characteristic	Continuing to smoke at 12 months	Self-reported smoking cessation at 12 months	Biochemically-verified smoking cessation at 12 months
Total (row %)	128 (58.7)	77 (35.3)	13 (6.0)
**Demographic factors**			
Age, median years (IQR)	50 (37.5 – 59)	50 (38 – 61)	56 (51 – 61)
Highest level of education attained, *n* (%)			
Less than primary	2 (0.6)	1 (1.3)	0 (0)
Primary	2 (1.6)	3 (3.9)	0 (0)
Lower secondary	52 (40.6)	32 (41.6)	6 (46.2)
Upper secondary	47 (36.7)	25 (32.5)	6 (46.2)
University degree, or equivalent, or higher	25 (19.5)	16 (20.8)	1 (7.7)
**Smoking-related factors**			
Median average number of cigarettes/day (IQR)*	20 (10 – 30)	20 (10 – 30)	10 (5 – 17.5)
Score on the Fagerström Test for Cigarette Dependence (IQR)*	6 (5 – 8)	6 (5 – 7)	5 (2.5 – 6)
Median years smoking (IQR)	22 (15 – 40)	30 (15 – 40)	30 (30 – 36)
Ever attempted to quit in the past, *n* (%)	92 (71.9)	60 (77.9)	9 (69.2)
Drink alcohol every day, *n* (%)	44 (34.4)	33 (42.9)	3 (23.1)
Drink caffeinated drinks every day, *n* (%)	95 (74.2)	50 (64.9)	10 (76.9)
Living with at least one other smoker, *n* (%)	32 (25.0)	17 (22.1)	3 (23.1)
Reasons given to quit, *n* (%)			
Personal health condition	123 (96.1)	73 (94.8)	12 (92.3)
Family’s health	15 (11.7)	11 (14.3)	2 (15.4)
Expense	8 (6.3)	1 (1.3)	0 (0)
**Quitting-related factors**			
Advised to quit by referral doctor, *n* (%)^†^	103 (87.3)	64 (90.1)	11 (100)
Received written material from referring doctor, n (%)^‡^	68 (57.6)	40 (56.3)	6 (54.6)
Days from baseline to target quit date, n (%)^§^			
Less than 14 days	87 (68.5)	62 (79.5)	11 (100)
14 days or more, or did not commit to a target quit date	40 (31.5)	16 (20.5)	0 (0)
Number of successful counselling phone calls, median (IQR)^§^	7 (5 – 8)	8 (7 – 8)	8 (7 – 8)

^*^63 missing values; ^†^18 missing values; ^§^Statistically significant (*p* < 0.05)

IQR, interquartile range

Results of the exploratory regression model of factors associated with self-reported smoking cessation are shown in Table 3. Smokers who had more successful counselling phone calls were more like to have quit (6 – 7 phone calls vs. five calls or less, odds ratio 7.46, 95% CI 1.53 – 36.24; all eight phone calls vs. five calls or less, odds ratio 12.17, 95% CI 2.87 – 51.69). We did a second model excluding Fagerström Test for Cigarette Dependence and average number of cigarettes per day, the two variables with a high proportion of missing values. The estimates in the second model were similar to the first model, except for days from baseline to personal quit date that became statistically significant in the second model. A personal quit date of less than 14 days from baseline were associated with a higher chance of self-reported cessation (odds ratio 2.23, 95% CI 1.06 – 4.67) when compared with ≥ 14 days or no target quit date. Because of the small number of biochemically-verified smoking cessation observations, a regression model was not performed to evaluate associations with confirmed cessation [22].

**Table 3:** Logistic regression showing factors associated with self-reported smoking cessation for at least 30 days at 12-month follow-up

	**Odds of self-reported cessation at 12 months**	
	**Model 1**	**Model 2**
AIC	192.903	273.855
Variables included in the model	Adjusted odds ratio (95% CI)	Adjusted odds ratio (95% CI)
Age, years	0.99 (0.95 – 1.04)	1.00 (0.96 – 1.04)
Highest level of education attained		
Lower secondary and less^*^	Reference	
Upper secondary	0.41 (0.16 – 1.04)	0.63 (0.31 – 1.30)
University degree, or equivalent, or higher	0.87 (0.27 – 2.76)	1.09 (0.42 – 2.81)
Average number of cigarettes/days	1.03 (0.98 – 1.08)	-
Fagerström Test for Cigarette Dependence		
Low (< 4)	Reference	-
Moderate (4–6)	1.44 (0.42 – 4.89)	-
High (7–10)	0.36 (0.09 – 1.46)	-
Years smoking (for each additional year)	0.99 (0.94 – 1.04)	1.01 (0.97 – 1.04)
Ever attempted to quit in the past	1.39 (0.50 – 3.85)	1.29 (0.62 – 2.66)
Drink alcohol every day	1.11 (0.47 – 2.59)	1.35 (0.71 – 2.58)
Drink caffeinated drinks every day	0.62 (0.24 – 1.61)	0.74 (0.37 – 1.50)
Living with at least one other smoker	1.22 (0.41 – 3.63)	0.92 (0.42 – 1.99)
Reasons given to quit (Yes vs. No)		
Personal health condition	0.47 (0.06 – 3.57)	1.17 (0.22 – 6.28)
Family’s health	0.69 (0.13 – 3.64)	2.08 (0.68 – 6.38)
Expense	0.21 (0.02 – 2.62)	0.20 (0.02 – 1.84)
Advised to quit by referral doctor	1.23 (0.31 – 4.89)	1.36 (0.46 – 4.00)
Received written material from referral doctor	0.97 (0.40 – 2.35)	0.83 (0.44 – 1.56)
Days from baseline to target quit date		
14 days or more, or did not decide a target quit date	Reference	Reference^+^
Less than 14 days	1.75 (0.69 – 4.43)	**2.23 (1.06 – 4.67)**^†^
Number of successful counselling phone calls completed by Quitline (maximum 8 calls offered)		
5 calls or less	Reference	Reference
6 – 7 calls	**7.46 (1.53 – 36.24)**^†^	**3.86 (1.28 – 11.67)**^†^
8 calls	**12.17 (2.87 – 51.69)**^†^	**6.98 (2.50 – 19.49)**^†^

^*^Combined due to small numbers in less than primary and primary levels

^†^Statistically significant (*p* < 0.05)

## Discussion

In the single-arm intervention study, we evaluated the feasibility of a smoking cessation intervention that recruited participants in three rural district hospitals in Vietnam. About half of smokers referred to the study Quitline were eligible and agreed to join the smoking cessation programme. More than half of participants answered all eight planned counselling phone calls. Two-third of participants made at least one quit attempt that lasted for more than 30 days. Around 40% of participants reported abstinence from smoking at the end of the study but only a small proportion of these self-reported quitters did urinary cotinine test. Overall, 5.9% of all participants achieved verified smoking cessation for more than 30 days 12 months after enrolment.

The smoking cessation rate in our study was comparable to prior studies involving similar interventions. A study with proactive quitline support found a 18.2% of self-reported prolonged cessation and a 7.7% verified cessation rate at 6 months among adult smokers who called a national quitline [23]. Another trial enrolling hospitalised adults also showed a similar result among those who received counselling calls after discharge (20% self-reported and 4.9% verified quit rate at 6 months) [24]. Another trial evaluating the effectiveness mobile phone text messaging found a 10.7% verified cessation and 19.8% of self-reported 28-day continuous abstinence at 6 months, compared to 4.9% and 13.5% in control group [25]. A number of other studies assessed self-reported cessation, without biochemical verification. A study of quitline counselling with translated languages enrolled Asian smokers in the United States [26]. Self-reported prolonged abstinence at 7 months was 24.3% among Vietnamese participants receiving counselling, compared to 15.6% among Vietnamese who received self-help materials only. In another trial, smokers enrolled in primary care who received tailored text messages had a self-reported 6-month prolonged abstinence of 15.1%, compared to 8.9% in the usual care group [27]. Self-reported quit rates at the longest follow-up in other trials with quitline or text messaging interventions ranged from 9.3% to 19.9% [28–31]. Our findings of a 5.9% verified cessation rate and a 40.7% of self-reported cessation rate suggested the intervention in our study is likely to be effective.

Quitline and text messaging are proven effective interventions to support smoking cessation. The low-cost nature made the two interventions suitable for widespread use, especially in resource-limited settings [11]. However, most Quitlines and text messaging require smokers to call or sign up first. In our study, the two interventions were combined as a smoking cessation programme operated by one Quitline centre, using existing infrastructure. The integration of the smoking cessation programme with brief advice provided by healthcare workers and referral from hospitals to a centralised call facility provides a model that could readily be scaled up more widely. A high retention rate and high participation rate in counselling phone calls also demonstrated the feasibility of the intervention.

We found an association between number of successful counselling phone calls and self-reported quitting. In existing literature, the association between planned number of calls and quit rates is inconsistent. A recent Cochrane review found some evidence that interventions with three to six calls may be more effective than those offering only one call [32]. The authors suggested further studies directly comparing different numbers of counselling calls to consolidate the evidence. As the actual number of phone calls delivered may differ from planned number, as in our study, we also suggest research evaluating the effect and potential dose–response of number of counselling phone calls on quit rate.

Despite the strength of the integrated intervention, we found a low proportion of smokers at healthcare facilities were referred by healthcare workers to the study Quitline. This could be explained by several factors. First, not all doctors were willing to take part in the study and refer their patients to our Quitline. Second, doctors may have not advised their patients to quit due to high patient loads in the clinics or their lack of confidence on smoking cessation [33, 34]. There is evidence that even very brief advice can improve quit rates [35]. Doctors should be encouraged to talk with their patients about quitting smoking, even in very short time, and provide available resources.

We found few healthcare workers volunteered to participate in the programme as smokers. Of those who did participate, 80% were not successful in quitting. A previous survey from three large hospitals in Vietnam showed a 35.6% smoking prevalence among male health professionals and 23% among doctors [36]. Healthcare professionals’ smoking behaviour may lead to less commitment to providing cessation suggestions, or less confidence in counselling [5]. Further studies are needed to evaluate the barriers to healthcare workers participating in smoking cessation interventions. Interventions targeted towards healthcare workers who are smokers should be considered in Vietnam, both for their health and for the benefit of their patients.

This study has some important policy implications. The Vietnamese government’s Directive 05/CT-BYT in 2013 emphasised the importance of scaling up smoke free hospitals [8]. This decision is supported by a guide for implementation, developed by the Vietnamese Committee on Smoking and Health, that has been piloted in nine hospitals across Vietnam [17, 37]. However, this policy has not yet been widely adopted. In our study, we encouraged the hospitals to strengthen smoke-free hospital, yet little was planned after study commencement. Further actions are needed to ensure proper implementation of Vietnamese regulations around smoking cessation within health facilities, and greater resourcing to support the smoke-free hospital policy.

The study has several limitations. First, a high proportion of participants who reported abstinence did not complete a urinary cotinine test at the end of study. Some of those who self-reported cessation may have not reported their smoking status correctly. Others may have refused due to the COVID-19 epidemic (99% of the study participants had their final contacts after March 2020). Second, it is possible that some infrequent smokers were misclassified due to the cotinine test strips, which give a single band result at a cut-off value. However, a false positive result due to environmental exposure to other sources of cotinine is unlikely, given the relatively high threshold of 200 ng/mL detected by the strips [38]. Third, although directors of the hospitals agreed for their staff to participate in the study, doctors were not required to deliver the intervention. As a result, recruitment to the study was lower than expected. Fourth, we were unable to implement a more comprehensive smoke-free hospital policy. Our research team will publish a formal qualitative assessment that was conducted recently on this topic. Finally, as this was a single-arm study, the effectiveness of the intervention cannot be determined. A cluster randomised controlled trial, with study design informed by this feasibility study, is currently ongoing to evaluate the effectiveness of the intervention in quitting smoking (ACTRN12620000649910).

## Conclusions

In conclusion, the integration of brief advice and referral from healthcare facility, Quitline counselling phone calls, and scheduled text messaging was feasible in rural hospitals in northern Vietnam. The scale-up of smoking cessation within hospitals, for both clinicians and patients, is an important priority within the local healthcare system.

## Supplementary Information

**Additional file 1. **

## Data Availability

The datasets used and/or analysed during the current study are available from the corresponding author on reasonable request.

## Abbreviation

LMICs: Low and middle-income countries
