# Supplementary material for: A smoking quitline integrated with clinician counselling at outpatient health facilities in Vietnam: a single-arm prospective cohort study
Source: BMC Public Health. 2022 Apr 13;22:739. doi: 10.1186/s12889-022-13203-y (PMC9006502; doi:10.1186/s12889-022-13203-y)
Supplement: Supplementary file 1 — Additional file 1. [file 12889_2022_13203_MOESM1_ESM.pdf]

Supplementary Table S1. Text message schedule

Day	SMS type	Tailored information	SMS content
1	Admin	Male	Dear Mr. NAME, welcome to VCAPS smoking cessation intervention. Congratulations on your decision to manage your smoking. We are here to help you prepare
		Female	Dear Miss NAME, welcome to VCAPS smoking cessation intervention. Congratulations on your decision to manage your smoking. We are here to help you prepare
2	Admin	Quit date	Well done for setting up your quit date on DATE. Let's prepare for it together!
3	Strategy		From now on, don't smoke inside your home or inside your car. Go outside if you want to smoke.
4	Admin		Managing smoking works best when you're prepared. Stick with us. We'll show you some strategies to deal with cravings and difficult situations over the next few weeks.
5	Strategy	Answered Yes to the question "Do you drink coffee/tea/colas every day?"	We know you love drink coffee/tea/colas. Try to cut the drink in half. It helps reduce your discomfort after you stop smoking.
		Answered No to the question "Do you drink coffee/tea/colas every day?"	Avoid drink too much coffee/tea/colas. It will help you manage your smoking.
6	Strategy	Answered Yes to the question "Do you smoke water pipes?"	Do you smoke water pipes? Water pipes are not a safe alternative. If fact, water pipes might be worse to your health than manufactured cigarettes
		Answered No to the question "Do you smoke water pipes?"	There is no such thing as safe tobacco. Hand rolled cigarettes, waterpipes, and cigars carry the same health risks as manufactured cigarettes.
7	Encourage	Male	Smoking causes erectile dysfunction. You have made a right decision to manage your smoking.
		Female	Smoking can cause many diseases, including cancer, stroke, heart disease, and blindness. Feel proud of yourself for deciding to quit.
8	Strategy	Answered Yes to the question "Do you live with a smoker?"	Remember the smoking outside strategy? It applies to everyone living with you. No one smoke inside!
		Answered No to the question "Do you live with a smoker?"	Share with your family that you are quitting smoking. Someone might be willing to support and encourage you.
9	Strategy	Answered 30 minutes or less to the question "How soon after you wake up do you usually have your first smoke?"	If you want to smoke right after you wake up, it means your body is special and needs more assistance. Don't worry because we are here.
		Answered more than 30 minutes to the question "How soon after you wake up do you usually have your first smoke?"	Do you believe you can stop smoking? We do because our trained Quitline counsellors are ready to assist you.
10	Encourage		Isn't it good to your family that you start to do something to manage your smoking? Everyone in the family will become healthier.
11	Admin		Remember you can calll the VCAPS Quitline at 1800 6276 if you need help

12	Encourage	Overall progress not so good, indicated by the counsellor after the 1-week call	We know it is hard but it is a decision you will not regret. Keep getting the support you need and remind yourself of your reasons to stop smoking.
		Overall good progress, indicated by the counsellor after the 1-week call	You are on the right track! Stop smoking is hard but stay confident. You can do this.
13	Strategy	Answered Yes to the question "Have you tried to quit smoking?"	Think about what strategies worked and what did not work well during your previous quit attempt. Use your past experience to help yourself.
		Answered No to the question "Have you tried to quit smoking?"	Be proud of yourself for deciding to stop smoking. Let's do it and make you a non-smoker.
14	Strategy		Think again your strategies to deal with cravings. If you don't have any strategy or are not sure about them, call the Quitline to get help.
15	Strategy		Knowing your triggers helps you learn how to deal with them. Talk to your Quitline counsellor and write down your top 3 triggers and your coping strategies.
16	Encourage		If you're feeling cranky it could be because you’re stopping smoking. This is only temporary. Call the Quitline if you need to talk about your mood.
17	Strategy	Answered Yes to the question "Do you drink alcohol every day?"	Would you like to reduce alcohol? It will help with your plan to stop smoking. And remember, don't smoke while you drink.
		Answered No to the question "Do you drink alcohol every day?"	It is good that you don't drink alcohol very often. Keep yourself away from alcohol.
18	Encourage	Overall progress not so good, indicated by the counsellor after the 2-week call	We know it is hard to persist. Believe in yourself and Keep getting the support you need to stop smoking.
		Overall good progress, indicated by the counsellor after the 2-week call	Good job! You are on the right track! Believe in yourself that you can become a nonsmoker.
19	Admin		Call the Quitline at 1800 6276 if you need assistance from the Quitline counsellor for dealing with cravings
20	Strategy	Answered Yes to the question "Do you roll your own cigarettes?"	Hand rolled cigarettes are not less harmful than ordinary cigarettes. They cause the same serious problems to your health.
		Answered No to the question "Do you roll your own cigarettes?"	Some people say hand rolled cigarettes are less harmful. It's not true.
21	Strategy	Answered Yes to the question "Are most of your friends smokers?"	It's not easy if you are surrounded by smoking friends. Can you find someone from your friends who also want to stop smoking?
		Answered No to the question "Are most of your friends smokers?"	Hang out with your friends in places where smoking is not allowed. It helps you manage your smoking.
22	Strategy		Urges for smoking often get away in few minutes. Get a quick exercise or eat a small snack.
23	Admin		Call the Quitline at 1800 6276 if you need help with your cravings or withdrawal symptoms
24	Strategy		Be careful when you go to a party or a smoking area. Do not let yourself slip. You have done so much.

25	Encourage	Participant indicated still smoking during the 3-week call	Stop smoking is difficult for some people. Let's keep trying. Call the Quitline if you are ready to choose a quit date.
		Participant indicated not smoking during the 3-week call	Congratulations on your progress! Keep your great work. We believe in you.
26	Strategy		Remember, no one can smoke inside your house or inside your car.
27	Encourage		You may feel strange when you stop smoking. This is withdrawal because your body is used to smoking. These feelings will go away in few weeks.
28	Strategy		Even the strongest cravings will go away after a few minutes. Focus on something else and remind yourself why you want to be smokefree.
29	Strategy		Researse beforehand in your mind how to resist if you are going to a place or party where people may offer you cigarettes.
30	Strategy		Gaining a few extra kilograms after stop smoking is normal. Eat healthy and exercise can prevent most of this weight gain.
31	Encourage		Can you see yourself as a nonsmoker? Trust yourself. You can make it happen.
32	Encourage	Participant indicated still smoking during the 4-week call	Stop smoking is difficult for some people. Let's keep trying. Call the Quitline if you are ready to choose a quit date.
		Participant indicated not smoking during the 4-week call	Congratulations on your progress! Keep your great work. We believe in you.
33	Strategy		If you are in a bad mood, talk about it with the Quitline counsellor or someone who supports you. Remember, your feelings matter.
34	Strategy		When you want to have just one cigarette, don’t think it's just one cigarette. Most people start regular smoking again after "just having one".
35	Encourage		Stay positive. Do not let things get you down. Your journey to a smokefree life might be a struggle, but looking back it will be well worth it.
37	Strategy		Don't let your friends smoke around you. Ask them to smoke outside or you can hang out with them in non-smoking places.
38	Encourage		Value your future. No matter when you quit, you are adding years to your life. You will not regret this.
40	Strategy		If you gain a lot of weight, go to see a doctor and seek medical advice.
42	Encourage		After you stop smoking, your lungs begin to improve and your heart attack risk begins to drop
44	Strategy		Practice in your head scenarios that might cause you to slip. Remember, do think it's just one cigarettes.
45	Admin		If you smoke again, call the Quitline at 1800 6276 to discuss the next step. Don't feel embarassed. We want to help you.
47	Encourage		Can you feel it? Urges are getting weaker and less frequent over time.
49	Encourage		Do you know how much money you spend on cigarettes? Think about what else you could do with that money.

51	Admin		We are sending less texts. But we are still here to help you.
53	Strategy		How well did your coping strategies work? Talk to the Quitline counsellor if your strategies were not helpful.
55	Encourage		Cigarettes never solved a problem for you. You did it yourself. You can do great things, so keep thinking positively
58	Encourage		There are so many benefits to being smokefree. What do you look forward to the most?
60	Strategy		No one smokes inside your house or inside your car. Anyone who wants to smoke should go outside.
62	Admin		Call the Quitline at 1800 6276 if you start smoking again. Our counsellor will discuss with you what we can do for you.
65	Strategy		How have you been feeling? Talk to your family or a friend if you are in a bad mood.
68	Encourage		You and your family are becoming healthier after you stop smoking. It's well worth it. Hang on to it.
72	Admin		Don't forget that you can call the Quitline at 1800 6276 if you need assistance.
76	Strategy		Don't forget your strategies for urges and difficult situations. Remind yourself and practice in your mind.
80	Encourage		Do you feel more comfortable and more confident after stop smoking? Feel proud of yourself for doing so much.
85	Admin		This is the last text. Good luck. You can still call the Quitline if you need support

Supplementary Table S2. Text message schedule around the target quit date

Day	SMS type	SMS content
1 day before	Strategy	Tomorrow is the day, you can do it and we are here to help. Review your coping strategies again.
Quit date	Admin	It's time to stop smoking. Call the quitline at 1800 6276 if you need any help.
1 day after	Strategy	Cravings to smoke only last for a few minutes. Take a quick walk or do a short exercise when you want to smoke.
2 days after	Strategy	Keep some sweet snacks in your purse or pocket. Take the snack instead of a cigarette next time you have a craving.
3 days after	Strategy	Avoid drink alcohol in the first 2 weeks will be helpful for managing your smoking.
4 days after	Strategy	Stress and anger are smoking triggers. If you're feeling stressed or upset. Call the quitline to get extra help. Or talk to your family or a friend.
5 days after	Encourage	"It always seems impossible until it is done." Never give up and don't be discouraged.
6 days after	Strategy	When you go out, choose non-smoking venues like the movies. This will help reduce your urges.
7 days after	Encourage	1 week smokefree! Do not look back now. Mark your calendar and do something special today to celebrate this milestone!

Supplementary Table S3. Comparison of characteristics by participants' smoking status at the end of study

Characteristic	Continuing to smoke at 12 months (Group A)	Self-reported smoking cessation at 12 months (Group B)	Biochemically-verified smoking cessation at 12 months (Group C)	P-value for statistical analysis*	P-value for pairwise comparisons
Total	128	77	13		
**Demographic factors**					
Age, median years (IQR)	50 (37.5 – 59)	50 (38 – 61)	56 (51 – 61)	0.2096	
Highest level of education attained, n (%)				0.9052	
Less than primary	2 (0.6)	1 (1.3)	0 (0)		
Primary	2 (1.6)	3 (3.9)	0 (0)		
Lower secondary	52 (40.6)	32 (41.6)	6 (46.2)		
Upper secondary	47 (36.7)	25 (32.5)	6 (46.2)		
University degree, or equivalent, or higher	25 (19.5)	16 (20.8)	1 (7.7)		
**Smoking-related factors**					
Median average number of cigarettes/day (IQR)*	20 (10 – 30)	20 (10 – 30)	10 (5 – 17.5)	0.4757	
Score on the Fagerström Test for Cigarette Dependence (IQR)*	6 (5 – 8)	6 (5 – 7)	5 (2.5 – 6)	0.0988	
Median years smoking (IQR)	22 (15 – 40)	30 (15 – 40)	30 (30 – 36)	0.6190	
Ever attempted to quit in the past, n (%)	92 (71.9)	60 (77.9)	9 (69.2)	0.5876	
Drink alcohol every day, n (%)	44 (34.4)	33 (42.9)	3 (23.1)	0.2735	
Drink caffeinated drinks every day, n (%)	95 (74.2)	50 (64.9)	10 (76.9)	0.3255	
Living with at least one other smoker, n (%)	32 (25.0)	17 (22.1)	3 (23.1)	0.8911	
Reasons given to quit, n (%)					
Personal health condition	123 (96.1)	73 (94.8)	12 (92.3)	0.7839	
Family's health	15 (11.7)	11 (14.3)	2 (15.4)	0.8341	

Expense	8 (6.3)	1 (1.3)	0 (0)	0.1675	
**Quitting-related factors**					
Advised to quit by referral doctor, n (%)†	103 (87.3)	64 (90.1)	11 (100)	0.4051	
Received written material from referring doctor, n (%)‡	68 (57.6)	40 (56.3)	6 (54.6)	0.9711	
Days from baseline to target quit date, n (%)				**0.0293**	Group A vs Group B = 0.1294<br>Group A vs Group C = 0.0193<br>Group B vs Group C = 0.0699
Less than 14 days	87 (68.5)	62 (79.5)	11 (100)		
14 days or more, or did not commit to a target quit date	40 (31.5)	16 (20.5)	0 (0)		
Number of successful counselling phone calls, median (IQR)	7 (5 – 8)	8 (7 – 8)	8 (7 – 8)	**<0.0001**	Group A vs Group B = 0.0004<br>Group A vs Group C = 0.0439<br>Group B vs Group C = 0.7208

*Chi-square test for categorical variables and Kruskal-Wallis test for continuous variables
